# Specialised residential care for older people subject to homelessness: experiences of residents and staff of a new aged care home in Australia

**DOI:** 10.1186/s12877-024-04791-y

**Published:** 2024-03-12

**Authors:** Costanza Preti, Christopher J Poulos, Roslyn G Poulos, Najwa L Reynolds, Allison C Rowlands, Kyall Flakelar, Angela Raguz, Peter Valpiani, Steven G Faux, Claire MC O’Connor

**Affiliations:** 1 Centre for Positive Ageing, HammondCare, Sydney, Australia; 2 UCL Institute of Education (Culture, Communication and Media), London, England; 3 School of Population Health, UNSW, Sydney, Australia; 4 Haymarket Foundation, Sydney, Australia; 5 The End Street Sleeping Collaboration, Sydney, Australia; 6 Departments of Rehabilitation Medicine and Pain Medicine, St Vincent’s Hospital, Sydney, Australia; 7 School of Psychology, UNSW, Sydney, Australia; 8 Neuroscience Research Australia, Sydney, Australia

**Keywords:** Ageing, Homelessness, Long-term care, Palliative care, Qualitative, Trauma-informed care

## Abstract

**Background:**

The number of older people experiencing homelessness in Australia is rising, yet there is a lack of specialised residential care for older people subject to homelessness with high care and palliative needs. To address this significant gap, a purpose-built care home was recently opened in Sydney, Australia.

**Methods:**

This qualitative study explores the experiences of both residents and staff who were living and working in the home over the first twelve months since its opening. Residents were interviewed at baseline (*n* = 32) and after six months (*n* = 22), while staff (*n* = 13) were interviewed after twelve months. Interviews were analysed using a reflexive thematic analysis approach informed by grounded theory.

**Results:**

Three main themes emerged: (1) Challenges in providing care for older people subject to homelessness with high care needs; (2) Defining a residential care service that supports older people subject to homelessness with high care needs, and (3) Perception of the impact of living and working in a purpose-built care home after six months (residents) and twelve months (staff) since its opening. A key finding was that of the complex interplay between resident dependency and behaviours, referral pathways and stakeholder engagement, government funding models and requirements, staff training and wellbeing, and the need to meet operational viability.

**Conclusion:**

This study provides novel insights into how the lives of older people subject to homelessness with high care needs are affected by living in a specifically designed care home, and on some of the challenges faced and solved by staff working in the care home. A significant gap in the healthcare system remains when it comes to the effective provision of high care for older people subject to homelessness.

## Background

The number of older people experiencing homelessness is rising [1] and statistical modelling predicts this to continue as the baby boomer generation ages [2]. The demographic of this social group consists predominantly of men, however there has been an increase in the proportion of women [3] and an over-representation of First Nations people [2, 4].

Research on the pathways leading to homelessness in older age indicates that complex interconnections between structural and individual factors are influencing this shift [5]. Among the structural elements, there is a lack of affordable housing [6], followed by economic instability where there are fewer available job options, and welfare systems that may not adequately support vulnerable populations [7, 8]. Individual factors include chronic mental health conditions, drug and alcohol use [9], family breakdown [10], a history of violence in the family, sometimes linked with non-heterosexual sexual identity [11], having been in the child welfare system, often as a result of physical abuse in the family [12], gambling addiction [13], and having spent time in institutionalised facilities such as detention centres, prisons, and psychiatric hospitals [14, 15].

Substance use, in particular, has been strongly associated with prolonged and persistent homelessness among older people with mental health conditions and has been described as one of the main causes of their premature mortality rates [16]. Furthermore, substance use among older people who are homeless is a growing public health concern and one that is linked with the ageing of the baby boomer generation, a cohort whose use of illicit drugs has been higher than any previous cohorts [17]. The literature suggests that due to the challenges experienced in their lives, homeless older adults usually ‘exhibit cognitive and physical characteristics of someone 10–20 years older and have a corresponding subjective age identity’ [18], which suggests that their physical and mental health closely resemble those of a much older person. Indeed, in both literature and practice, the age of 50 has been used as a threshold to typify ‘older’ homeless persons [19]. Older people experiencing homelessness present with unique health, psychosocial and geriatric problems (e.g. mobility impairment, falls, frailty, cognitive impairment and urinary incontinence) that are difficult to treat without housing stability, ongoing health support, and a care plan in place, often leaving this cohort with persistent and deteriorating health conditions [20].

At present, there is a lack of specialised residential care for older people subject to homelessness with high care and palliative care needs [21, 22], a significant gap in Australia’s aged care system. In Australia, a complex aged care system that involves different legislation across both federal and state governments contributes to confusion and service gaps where different models of care coexist [23]. Additionally, there is limited provision of funding for aged care providers to support people who are experiencing or are at-risk of being homeless (Beer et al., [23]. Homeless and older people who are at-risk of being homeless are addressed in social policies where the shortage of social housing has been described as one of the main factors that contribute to homelessness in the Australian society [24, 25]. When it comes to accessing residential care, older people experiencing homelessness in Australia have a limited choice of options such as shelters, transitional accommodation, and crisis accommodation (e.g. temporary shelter, residing in boarding houses or couch surfing), which do not provide nursing care [26]. In addition, such facilities are not equipped to provide specialist assessment and behavioural support, an aspect that is important to address the complex needs of this cohort of people [27].

To address this gap, a new care home for older people with high level needs was opened in March 2020 in inner-city Sydney, as this area presented an uniquely high proportion of older individuals subject to homelessness [28]. The new care home has adopted a trauma-informed model of care where staff are specifically trained to care for a cohort of residents with traumatic lived experiences, and associated complex physical and psychological needs [27, 29]. The health, wellbeing, and cost-benefit outcomes for residents over the first 12 months of living in the home have been published elsewhere [30]. In brief, resident scores in personal wellbeing and overall health-related quality of life improved, and levels of frailty, physical functional independence and global cognition were maintained over the first 12 months of residing in the home.

The aim of the present study was to: (1) understand resident and care home staff perspectives on their lives in the new home; (2) explore the challenges faced by both residents and staff over their first 12 months of being in the care home and (3) outline implications for the replication of similar services.

## Methods

### Study design

The study was part of a larger service evaluation of a new care home in Sydney (Australia) targeting older people subject to homelessness. The main body of work [30] defined people as being ‘subject to homelessness’ if they are/were homelessness, or if they were ‘at-risk’ of possible homelessness. This aligns with a recent report by the Australian Housing and Urban Research Institute highlighting that strategies to address homelessness must include people who are homeless and at-risk of homelessness [1, 31], therefore, the term ‘subject to homelessness’ was used to describe the cohort in the present study. The evaluation used a mixed-methods approach to explore quantitative health and wellbeing outcomes over the first year of operation of the care home, together with costs and cost benefit [30]. This current paper presents the qualitative data from the perspective of residents and staff who were living and working in the new care home. For the residents, this included their views on living in the new facility, and the consequent perceived impact on their health and wellbeing.

### Setting

The care home was designed ‘to provide a sense of community, foster a sense of dignity and facilitate independence and movement’ in the context of providing high level residential aged care [32], through 42 individual rooms with ensuites and balconies, over four floors. One floor was exclusively allocated to female residents and each floor has a shared lounge, kitchen, dining and quiet area [29]. The care home was built as a partnership between the not-for-profit aged care provider and a church in the inner city of Sydney where homelessness is concentrated, with funding support from the provider, private and other philanthropic donors, and from Local Government [32; Personal Communication]. Extensive consultations with the age care service sector and local homeless service providers in the area were also held prior to the building stage [29]. The vision of the new facility was to provide a high level of care for older people who are homeless or at-risk of homelessness with complex care needs. The top floor of the facility was reserved to palliative care residents, to provide a dignified end of life care to a cohort that tend to die alone and apart [32]. Through both the design and operation of the home, a trauma-informed approach to care was considered [30]. The facility had a nurse on the premises 24/7, and specially trained staff, including palliative care specialists, and pastoral care workers. Additionally, a care-worker led staffing model facilitates trust development between staff and residents. In order to live in the home, residents are required to pay a fixed percentage out of their government-funded aged pension.

### Participants and recruitment

Residents who were admitted permanently to the care home were invited to take part in the study. Residents who were admitted for ‘respite’ on a temporary basis were not recruited. In order to participate in the study, residents were required to be willing and able to give informed consent and comply with the study requirements (for the purpose of this study it was to participate in interviews). It was made clear to each resident that participation in the study was completely voluntary and that non-participation would in no way affect their residency within the home. The researchers applied the tenets of supported decision making [33] to assist participants in making an informed choice about participating in the research.

The recruitment of staff working in the aged care home was done initially through flyers outlining the purpose of the study, that were placed in communal staff areas. Some of the staff approached the researchers directly while others were approached by the researcher and agreed to be interviewed. A convenience sample was stratified according to the staff professional role to ensure a diverse cross section of experiences were represented (e.g., nurse, carer, pastoral care, manager, admission staff, etc.) and different shifts (morning, afternoon, night). Identified staff were contacted by email with a participant information sheet and informed consent was obtained prior to being interviewed. The study was approved by the St Vincent’s Hospital Sydney Human Research Ethics Committee [2019/ETH11898].

### Data collection

One-to-one semi structured interviews were conducted with the participants by an experienced team of qualitative researchers (AR, NR, COC, CP), either face to face within the aged care home, or via online video call. Resident interviews took place between March 2020 – April 2021. Residents were interviewed at baseline, being soon after admission to the home, and again after six months, to explore their personal history leading to homelessness (or being at-risk of homelessness) and their experience and perceptions of the changes in their lives resulting from living in the care home. Staff were interviewed between March – May 2021, a year after the care home had opened, to explore their experiences of working within the care home. Interviews were audio recorded, de-identified and transcribed verbatim by an external transcription company. Of note, this study was conducted during the COVID-19 pandemic, and during this first year of operation the home was subject to periods of lockdown which may have impacted on resident and staff experiences.

### Data analysis

Transcriptions were analysed using a ‘reflexive thematic analysis’ approach [34, 35] informed by grounded theory [36] as the interview questions were not underpinned by an hypothesis, but their nature was explorative. The reflexive thematic approach allowed the research team to identify patterns through the data, to understand participants’ perceptions of their experiences [34], and grounded theory supported the development of the analysis without theoretical constraints [37].

The research team who collected and coded the data had experience in conducting social science research, with a specialisation in dementia care, aged care, rehabilitation, occupational therapy, social work and experience in research in hospitals. However, they did not have previous experience of researching older people experiencing homelessness. Through regular meetings, shared notes, and open communication, the team discussed their daily experience in the care home and the nature of their interactions with the different participants. These formal and informal discussions informed the data analysis which was a rich dataset including participants with different roles (residents and staff) which helped the triangulation of data.

Content analysis of the transcripts followed the six steps outlined by Braun and Clarke [34]: (1) familiarise with the data through reading of the interview transcripts; (2) generate initial codes; (3) search for themes; (4) review themes while (5) generating thematic maps of the dataset; and finally, (6) define a final list of themes through a refining process that connects themes through causal relationships. Codes were grouped into sub-themes and subsequently grouped under overarching themes which represented a central definition [35]. Interview transcripts and interview notes were imported into Atlas.ti (version 9) [38] and initially coded by one researcher (CP). Consistency was enhanced through inter-coder reliability checking with another member of the research team (NR) to minimise subjective bias [39]. This process followed the steps highlighted by Nili and colleagues [40] where, after discussing a coding scheme, a fellow researcher coded transcriptions of selected interviews. This phase was followed by two separate meetings between the researchers (CP, NR) to discuss inter-coder reliability for segments of text coded and to revise the final list of coding.

## Results

Residents taking part in the study were interviewed at baseline (*n* = 32) and after six months (*n* = 22). Due to their high health care needs it was not always possible to interview all participants recruited at each time point; 10 residents were interviewed at baseline only, while 22 residents were interviewed both at baseline and at six months, and two residents were not able to be interviewed at either time point. The demographics of participants are presented elsewhere [30]. In brief, the residents taking part in the study had a median age of 75.6 years, the majority were males (65.7%), and most were considered to be ‘at-risk’ of homelessness (62.9%), while just over a third of participants had experienced homelessness at some stage of their life (37.1%). Prior to their admission into the care home the majority of residents were living in Government housing (57.1%) and some were previously in lower care residential care (20%). Most residents were referred from a hospital (62.9%), followed by other care providers (25.7%) and homelessness services (8.6%), with one resident referred by a friend. The majority of residents moved into the home due to a range of high-level care needs that spanned two broad categories: (1) high health care needs (54.3%) such as functional decline and inability to manage independently, or (2) high care needs due to psychological, cognitive, or substance-related support needs (40%).

Staff (*n* = 13) were interviewed 12 months after the care home was opened. The majority of care home staff were female (61.5%), with the most represented age group being 25–34 years (53.8%). Staff predominantly identified as Australian (69.2%). The majority of staff who were interviewed did not have prior experience working within the homelessness sector (84.6%) (see Table 1). There was a range of experience across staff in terms of number of years working within the aged care sector, and all staff had been working within the current organisation for over six months.

**Table 1:** Staff demographics (*n* = 13)

Sex	Count (%)
Female	8 (61.5)
Male	5 (38.5)
**Age** (years) 18–24 25–34 35–44 55–64	**Count (%)** 1 (7.7) 7 (53.8) 4 (30.7) 1 (7.7)
**Prior experience working in the homelessness sector** Yes No	**Count (%)** 2 (15.4) 11 (84.6)
**Self-identified cultural/ethnic background** Australian Nepalese Chinese African	**Count (%)** 9 (69.2) 2 (15.3) 1 (7.7) 1 (7.7)
**Duration of working experience in aged care** Median Interquartile range (25-75th percentiles)	**Years** 3.5 1–7
**Duration of employment with the current home** Median Interquartile range (25-75th percentiles)	**Months** 11 7–15
**Roles**	Administration officer Carer (afternoon shift– Level 1) Carer (morning shift– Level 2) Carer (morning shift– Level 3) Carer (night shift) Manager Manager Manager Occupational Therapist Pastoral Care Registered Nurse (morning shift) Relief Manager Volunteer Coordinator

Themes were derived by the combined analysis of resident and staff interviews. Table 2 provides a detailed overview of the identified concepts and subthemes; the final themes are presented in the central column, while on either side of the table are the contributing sub-themes identified in the resident group (left) and staff group (right). Three main themes emerged:

1. Challenges in providing care for older people subject to homelessness with high care needs.
2. Defining a residential care service that supports older people subject to homelessness with high care needs.
3. Perception of the impact of living and working in a purpose-built care home after six months (residents) and 12 months (staff) since its opening.

**Table 2:** List of final themes and subthemes according to resident or staff interview data

RESIDENTS were interviewed at baseline (*n* = 32) and 6 months (*n* = 22)		FINAL THEMES	STAFF (*n* = 13)	
Main Themes	Sub Themes		Main Themes	Sub Themes
Life before coming to the nursing home	• Living in different accommodations (e.g. shelters, boarding houses, hostels) • Professional life • Living in Social housing • Living a homeless life • Living in different nursing homes	THEME 1 Challenges in providing care for older people subject to homelessness with high care needs	Challenges in the job	• Resident behaviour: o Additional roles needed o Additional resources needed o Good support received within the organisation • English as second language - Barrier for in depth conversations
Events that could have determined the shift to homelessness	• Drinking problems • Break up in the family • Let down by the system • Reasons why people choose to live a homeless life • Suggestions to end homelessness			
Family and social connections	• Family link • Social links with friends			
Paths of referral to the nursing home	• Reasons behind their referral: health deteriorated • Who helped with the transfer to the nursing home • Path of referral - from hospital [to respite] to a nursing home	THEME 2 Defining an aged care service that supports older people subject to homelessness with high care needs	Referral mechanisms	• How are the admission criteria supporting homeless people • Finding the right match between new residents and current residents • No appropriate referrals from homelessness agencies • How are the residents assessed before being accepted • The main feeder is the hospital • Admission criteria have been tweaked since the opening • The aim is to target high care and palliative care needs • Relations with external services (hospital, agencies)
			Themes that have been discussed but still need to be clarified	• Defining the aims of this service within the organisation • Debate within the organisation about the service offered and its targets
Life after coming to the nursing home	• Positive comments about living at the home - after 6 months • Life after coming to the nursing home - expectations not met • Interaction with other residents • Activities in the home • Health improved • Expectation met • Financial issues • Sense of purpose	THEME 3 Perception of the impact of living and working in a nursing home after 6 and 12 months (respectively) since its opening	Reward, motivation, satisfaction	• Rewarding aspect of the job • Motivations to stay in the job • Satisfaction with the job
Interactions with staff	• Positive views of staff • Negative views of staff • Cultural differences of staff		Supporting the residents	• Supporting the residents’ sense of purpose • Spiritual care
Living in a nursing home - perspectives	• Different perspectives around food • Comments about living in a nursing home - expectation and reality • Residents’ personal goals • Suggestions for improvement		Working with homeless residents - what makes a good carer	• Personal quality needed in the job • Additional training identified • Training vs. no training received • Prior experience vs. no prior experience

### Theme 1: challenges in providing care for older people subject to homelessness with high care needs

Before their referral to the home, some residents reported living in accommodations that may not have met basic standards of hygiene (the transcripts include accounts of the presence of insects and rodents) and where they could not manage to live independently due to their increased care needs. Most residents had been accommodated in government owned social housing where the resident had lived for a long time. Some residents reported that they had to live on a higher floor and without a lift, and due to their decreased mobility, had difficulty moving in and out of the accommodation:

[…] the stairs [were] my main obstacle and the fact that I can’t breathe. The building is filthy, it’s disgusting, it’s mouldy it’s - it’s falling down around me literally. I walked downstairs the other day and a whole chunk of cement fell on my head. And that’s happened three times. [R21]

Residents who moved into the care home from lower care facilities, such as other aged care homes, hostels and boarding houses, described *‘shocking’* hygiene issues, crowded rooms, poor facilities and incidents where staff were perceived to steal money from the residents. The quote below is from a resident who was coming from a fully concessional residential care home for people who are homeless or at-risk of homelessness:

One of the staff borrowed money off me and stupid me, I gave this person money. That caused a lot of drama and then the manager found out about it because somebody dobbed […]. We had a four bedroom, four people in our room, but it was a big room […]. I had one bloke [name], he was fantastic, but the other bloke was just, you know, he was just obnoxious really. He stayed all night with his TV up and I don’t know, dramas, dramas. Then I’d say turn your TV down and that. In the end the manager got him an ear plug. [R29]

Drinking addictions and family break-ups were reported as significant events that might have caused a shift in their lives:

I stayed there for about 2 years and then I started drinking again, I was going downhill fast. So I’ve - what I done - that’s right, the relatives sold all the places, all the property. So I - what’d I do - I went to - got a Housing Commission [government housing], that’s right […]. That’s where I went there and I had a few girlfriends and bit of a rough life there, you know. On drugs and shit, you know, alcohol. So I sort of woke up to myself and I chucked the place in and started just sleeping on the trains. I thought this is good this, you keep all your money, you’re not paying nothing and it’s not too bad. I didn’t smash the place up, and they’re watching all the time, the railway people. I was homeless for um - what year is it now? [R10]

Some of the residents discussed a traumatic childhood, mostly spent in institutions and did not have any family support around:

I’ve been in government custody I suppose you’d say, as a kid […] Not staying with people, but in foster care, I’m talking about staying in kids’ institutions. I was in and out of those all the time. [R21]

While others had few family connections they were able to maintain over time, although they often discussed taking care of children that were following a similar path (drug addiction, homeless, mental health issues):

My son comes occasionally. He’s got schizophrenia and he can’t sit for five minutes. He comes in and says hello and then he goes. At least he comes. [R15]

Despite the specific trauma informed training that the staff had received prior to their employment, navigating the varied behavioural challenges that residents presented as a result of their past, was described by the staff as a difficult aspect of their job. For example, some residents demonstrated *‘guarded’* behaviour and an unwillingness, or inability, to trust the staff for medical treatment/care. The personal background of the residents and the trauma informed model of care implemented in the home meant that the residents’ decisions were considered paramount, even if that resulted in not pursuing specific treatment if it was refused. This aspect was considered challenging for some of the staff as their professional training and the trauma-informed approach promoted in the home were often hard to reconcile:

Not everyone is willing to accept help or willing to want to get better. Even though you might explain to them the benefits of doing a certain practice, they’re still not interested. […] [Name], for instance, he could have lost his foot at one stage if he wasn’t careful, but he still wasn’t interested in attending an appointment with the High Risk Foot Clinic, and he wasn’t interested in us dressing it and cleaning it regularly. He’s better now, but the fact that he understood when we explained it to him when he was sober, that he could potentially lose his foot, it still didn’t click. He still wasn’t interested in anything to make it better. And so I think there’s a few people like that that they’re not interested in doing something to, that they know better their health. They just want to live. They just want to sit around, do their own thing, be their own person, and still have that independence of choosing that. “No, no don’t want to do that.” Which is completely different to a lot of other services […] I’d like it just a bit more guidance in how to manage people that refuse medical care. [S04]

Some of the staff were wondering whether this model of care could have included a ‘recovery’ element where residents were supported through actively processing some of their past trauma:

I think the discussion is worth having about you know under the heading of “recovery” really. Like, you know, everyone- most people have some trauma. A lot of people have mental health. Some people have background in addiction. And, you know, is there a role for us to work more proactively and deliberately in that space? […] we’re not any other facility because of this specific cohort you know, people with trauma and a homelessness background. So do we need to be more intentional about thinking about helping people to process trauma or, anger management or addiction issues or, in the more kind of what I loosely call a recovery area. [S07]

However, the trauma-informed model implemented in the new care home was perceived as quite different by some of the staff who struggled to reconcile the theoretical premises of the approach with the more traditional model of care they were used to:

What I didn’t really expect is the fact that people, the residents here are allowed to smoke. Most of the aged care where I have worked before people don’t smoke or the cigarettes are rationed. […] And another one is that some residents use actually drugs here. They cannot admit it, but they use them, like we had one who overdosed himself last week, so those are the issues that I thought maybe they are going to do like a rehabilitation or something […] things are different to this. But it’s very difficult here. [S10]

The challenges in dealing with the residents’ behaviours were also expressed, with staff noting that they often had to learn from experience about dealing with behavioural triggers in residents, and would have liked additional training, such as in the areas of mental health.

So training on how to adapt to different behaviours or people’s triggers or what that kind of thing would be beneficial. [S03]

Most of them don’t want to talk about their families. Just the mention of family for them, some of them it’s a trigger. So yeah, so we avoid talking about it unless there are ones who talk about their families, yeah. [S10]

### Theme 2: defining a residential care service that supports older people subject to homelessness with high care needs

Managers spoke about how the admission criteria for residents impacted the care home. Admission criteria involved two key factors: (1) the resident being ‘subject to homelessness’, and (2) the care needs of the resident, which determined the level of government funding provided (based on the Aged Care Funding Instrument (ACFI) and a ‘homelessness supplement’) [41], which in-turn impacted the ability of the care home to meet its operational requirements [41]:

For us we do have the homelessness payment that we get from the government. There are four areas to meet that criteria for the Homelessness Supplement […] they have to be homeless or at risk of homelessness. They need to be either living in temporary accommodation, in a boarding house or in a housing commission or obviously on the street or at a friend’s house. They also need to require support either with behaviours or activities of daily living. And they do need to have a particular diagnosis. Usually a mental health diagnosis or a dementia diagnosis to fit that criteria. If they don’t fit that criteria then we don’t claim the Homelessness Supplement and the issue there is sometimes they’re too low care to be here. On the flip side, if they don’t meet the Homelessness Supplement but they are high care for our ACFI then we usually will accept them as well. [S12]

The admission criteria were difficult to juggle on both a financial level and a social/organisational level. If the admission team was accepting more residents who were homeless or at risk of homelessness, but with lower care needs, they needed to ensure a safe environment for the other residents living in the home:

it will be great to be able to start getting more people in that are more at that end stage of life and palliative care where they need more higher nursing care and I think as we go, we’ll define that better but it’s challenging with the criteria that the government expects you to have with that homelessness [criteria]. Keeping that criterion and keeping the space safe is a very fine line. I think we’ve got there but it still comes with challenges. [S11]

Over the first 12 months of operation, the management team was refining the admission criteria, getting an understanding of the scope of the service and outlining some of the limitations needed to be in place to create a functional service:

So we can’t take people who have those violent tendencies which you can see in a lot of mental health issues. We really were meant to be for that end of life palliative care or higher complex nursing care, not higher complex mental health issues. [S11]

The most common referral pathway for residents was from a hospital, and these residents tended to have high care needs. Staff felt that this resulted in a better match between the needs of residents and staff in the new home, and over the first 12 months of operation a greater proportion of residents were following this referral path:

We have achieved full capacity twice in the last five months so that’s really good. We are getting a lot of referrals that are suitable. We still do get a few that aren’t suitable but we are feeding that back to the external stakeholders, so the hospitals, for example. Because we aren’t a secure site so we can’t take those residents who are wanderers and who are significantly cognitively impaired. Not knowing time and place, we’re not designed for that. [S12]

By 12 months, the management team had strengthened the relationship with different hospitals in the area and had put in place a referral system to ensure a high occupancy:

Most of our referrals now are coming from the hospitals […]. The improvement is that we’ve now got the referrers contacting the service a couple of times a week to check what vacancies there are. So I think the effort in building relationships with these referrers have actually strengthened. There’s more clarity around what we’re doing and that’s been a significant reason why occupancy has gone up. [S13]

The staff also reflected on the relationship with community homeless organisations who had high hopes for the opening of the new facility due to lack of suitable accommodation for older homeless residents [27]. This resulted in disappointment for community homelessness services, when they were not able to gain access to the care home for their clients when the admission criteria could not be met:

We’re not a low care hostel like [name] and [name] and I think it’s really important for the agencies to understand that so they don’t feel like we’re pushing back on them […]. I think what they’re expecting is that we’re sort of like the [hostel] and the other services but our space was meant to be different. We were supposed to be taking those that they weren’t able. I guess the challenge is that where they can’t get people in is where people are living with significant mental health, aggressive violent behaviours and that’s not our service. [S11]

### Theme 3: perception of the impact of living and working in a purpose-built care home after 6 months (residents) and 12 months (staff) since its opening

When asked about the perception of their lives in the care home, the residents frequently cited benefits, such as living in a private single room with a balcony, common areas where food was prepared for them, a 24-hour nurse on the premises, and night staff to respond to their requests and needs:

It’s great. It’s the right size. Got a nice big balcony. There’s an ensuite so I can, you know, go to the toilet myself. I can wash myself. I’ve got remote TV and DVD player. I’ve got a guitar in there. I practise sometimes. So yeah, I’ve got everything I need really. [R13]

Residents also reflected on how the new living arrangements had changed their regular habits and behaviour:

If I was on my own I’d probably start drinking or drugging or who knows. See it’s different now like I mean I’m in a good stable way now, like going to bed, waking up. It’s proper you know like everything’s good. So that’s good like, everyone says it’s very expensive but you pay [a government determined fixed percentage of the aged pension], it’s like anything, you get an old car, fifty dollars mate, that’s what you’re driving. But if you want to drive a Mercedes you’ve got to pay for it. [R10]

Some residents reported a drop in their drinking habits, something that they associated with living in the new home:

Resident: I did drink and I did smoke. But I don’t do it now. And my drinking has gone right right down but I still have a couple now and again. I ask though. But about that, that’s about it. [R01]

In addition to appreciating the ‘open-door’ policy of the home where residents are free to come and go as they wish, residents reported a sense of freedom, connected with feeling safe, supported and secure in the new facility:

Two people here every day, looking after you […] and these other ladies come in all the time. People here are that sick sometimes they can’t feed themselves, people are that good that they sit there with them and feed them […]. If I have to go to hospital or anything they’re ringing up checking on me you know, yes when you coming back you know they come and see you or something you’re not alone [R10].

My health has certainly improved. My perception has improved. By that I mean I am more aware around things going on and so on. I’m more what’s the word, where’s the word, I’m grateful? [R07]

Residents also reported a new sense of purpose that they had developed since living in the care home and possibly connected with living together with other people in a safe environment:

Interviewer: In the last six or so months that you’ve been here, do you feel like there’s been any changes for you?

Resident: I feel like I’m needed […] Instead of being by myself all the whole time.

Interviewer: In what way do you feel needed?

Resident: Just things that go on here. […] People I talk to at tea-time whatever I just feel like I’m in a place where I should be. [R14]

The resilience of the residents in facing the challenges of their complicated lives and their compromised health, appear to be one of the main reasons that motivated the staff to work in the facility:

People’s resilience is pretty astonishing. You know one guy who lived rough for seven years, he’s very, very sick so he has to negotiate lots of hospital visits and stuff and he finds it all incredibly intimidating. But he has tremendous resilience to keep going back keep turning up keep trying to get better, get his health under control when it would be easier to give up there. So people’s resilience is amazing really. And I think sometimes we don’t honour that enough. Like one guy who has never met his son. Ever. And he said to me, a few months ago, “Oh my son is turning 51 this month.” It broke my heart you know. So he’s lived his whole adult life with a major mental illness, knowing he’s got this son somewhere in the world. So a sense of loss, bereavement around that. And yet he keeps going, keeps trying to be kind to people you know, human resilience is just astonishing really. And I think we don’t celebrate that enough with that. [S07]

Furthermore, the residents expressed gratitude and an overwhelmingly positive view of the staff, who were described with superlatives such as *‘excellent’*, *‘fantastic’*, and *‘out of this world’*. The staff in turn reported feeling rewarded by the residents’ appreciation:

When I see changes and the positive changes in a person, what you did for them, that’s motivating. Yeah. And when people appreciate your work, that also motivates you. [S08]

Some staff felt that a more diverse range of activities and outings could have been offered to the residents to break up the daily routine and provide greater opportunities for residents to set their own goals. Residents, likewise, also suggested the provision of more and diversified activities (both residents and staff mentioned the idea of going out with a hired bus). Notably, during the study period, the COVID-19 pandemic limited the activities and outings that were possible due to restrictions placed on the care home residents and staff by the COVID ‘lockdowns’. Overall, the perception of the first six months living in the care home was positive for most residents, however, some used more critical terms to describe their situation. This was often associated with a deterioration in their health and of the personal struggle associated with a sense of loss and uncertainty:

Resident: […] I’m going demented. I’m just petrified about everything.

Interviewer: What kind of things are you most frightened about?

Resident: Well, not being in charge of anything; Never knowing if you’re going to be worse off or not and the age of myself. [R26]

Individual behaviours and the interaction between residents were described as a potential source of tension by the staff. Some conflicts between residents were recounted by the residents, sometimes due to money that was borrowed and not returned, other times when residents were apparently drunk and interacted rudely with some of their fellow residents. Such behaviours were reported to impact the residents’ perception of their quality of life in the care home and created some frustrations among the residents:

It’s fine for them [other residents] to go and get drunk as can be, come back here and we’ve all got to suffer because of it. You know that’s not right, that’s not right. I was told that there’d be a level of abstinence but from what I see […] [R04].

And a similar account, highlighting the tipping point of an already strained relationship between co-residents where vulnerability and potential exploitation can result in sudden aggression:

So I give him $40 and said go and buy a packet of smokes and he did and a couple of weeks later he come back for more. Said “can you give me $30 so I can go and buy a packet of smokes?” So I’ve given him $30. Yes I said “But this time you’ve got to pay me back”. and he come back in another fortnight… and he asked for another $40…and I said but you won’t get no money more out of me unless you pay it back. He never paid the $60 back. So I don’t lend him no more money. And I threatened him on the smoking verandah…. I went to smash his face in cause he turned the light on. And I don’t like the light on…in the dark and…I threatened to bash him…Yes. except for um the bloke [name] with me, here in this room.and I would have bashed him and thrown him over the verandah. That’s how mad I was. (R06)

## Discussion

The purpose-built care home that opened in inner city Sydney in March 2020 is one of the very few designated care homes for residents with high care needs who are subject to homelessness in Australia [42] and internationally [43]. In parallel with the broader service evaluation [30], this qualitative study explored the experiences of living and working in the home over the first year of its operation from the perspective of both residents and staff. The discussion is presented according to the themes identified in the results section.

### Challenges in providing care for older people subject to homelessness with high care needs

The different accounts that the residents provided about their previous lives, in terms of their housing situation, relationships, and physical and mental health, largely reflect findings reported in the literature [9, 22, 44, 45], suggesting that this cohort of residents exemplified the experiences of a reasonable cross section of an ageing population of people subject to homelessness. The staff identified the complex behaviour of the residents as one of the main challenges of their job. While many staff had experience working in aged care homes with people living with dementia who experience dementia-associated behaviours [46], the complex behaviours exhibited by the residents in this new care home were different, for example, associated with substance abuse or previous life trauma, which aligns with the reported characteristics of this cohort [45, 47]. There is also potential that some of the residents had dementia in addition to their history of being subject to homelessness [45, 47]. The relationship between homelessness and dementia appears to be a multilayered relationship, as suggested by Babulal et al. [48], whose review of the literature linked homelessness as a risk for and consequence of Alzheimer’s disease and related dementia, but also as a co-occurrence with psychiatric health, substance addiction and traumatic injuries.

The new care home adopted a trauma informed model of care where residents were able to live in the care home without any restriction, in line with the principle of safety, choice, collaboration, trustworthiness and empowerment [49]. Although staff had received specific professional training focused on trauma informed care, a model of care that respected the residents wishes (including the right to refuse care) above other health-related needs, they reported some difficulties in reconciling this with their previous general aged care training. While some staff reported feeling challenged when it came to the conflict between care which they felt was required to support a resident’s optimal health, and a resident’s refusal of that care, the Australian Charter of Aged Care Rights clearly states that all care recipients have the right to have control over their care, even if it involves personal risk [50]. However, in Australia, where a resident does not have capacity to make informed decisions and they do not have family or friends who are able to act on their behalf as guardian, there are independent state-based entities that formally appoint a guardian to ensure that the resident’s welfare, interests and views are considered and supported (NSW Government, [51]) For the present study, whether or not a resident was deemed to have capacity to provide informed consent and therefore participate in the research interviews was determined via the referring agencies and Aged Care Assessment Team during the process of admission to the home.

Currently there are no formalised guidelines of care delivery for older people subject to homelessness, and the delivery of care is experienced-based [16]. The literature reports that staff working in homeless settings receive minimal training in dealing with difficult situations, resulting in staff burnout, emotional exhaustion, and high turnover [52, 53]. While in the current study staff reported struggling to cope with aspects of resident behaviour, these challenges seemed to be offset by the rewards that staff experienced when they felt they were able to make a difference in the lives of the residents, who were in turn grateful and often expressed their appreciation to the staff. The reward experienced in the job was an important aspect that motivated staff and contributed to their overall job satisfaction. Olivet and colleagues [54] reported similar findings when they investigated a collaborative initiative to help end chronic homelessness in the US. Staff in this US study reported good levels of job satisfaction regardless of whether they experience high level of stress with associated mental health problems.

### Defining a residential care service that supports older people subject to homelessness with high care needs

From a more practical perspective, this evaluation also explored issues around the resident admission process. Overall, the recruitment process appeared to be a highly balanced process where health, social, behavioural, interpersonal, as well as economic and ethical factors had to be evaluated before a decision was made about which resident to accept. Findings suggest that staff were challenged when it came to balancing strict government requirements for residents to meet ‘homelessness’ criteria (greater than 50% of residents had to meet this requirement), government funding mechanisms based on resident dependency, the capacity of the home to support residents with a range of health and mental health needs, and the need for the care home to operate within its budget. Indeed, the results highlighted the challenges around meeting this balance. For instance, while a resident may have had a diagnosis of dementia or a complex health condition and met criteria for high level care, they may not have been able to be accepted for admission if they also had severe behavioural challenges, as the care home did not have a locked unit. Also, there was sometimes frustration expressed by community based organisations who were hoping to find accommodation for their homeless clients when there was a mismatch between their expectations and that of the care home staff around client suitability [27]. Indeed, only a third of residents in this study had a history of homelessness as opposed to being ‘at-risk’ of homelessness [30]. Thus, meeting the expectations of all community stakeholders was both a challenge and point of friction for staff. Clearer government funding guidelines which aim to support the future development and viability of care homes catering specifically to the growing older population could remedy this situation.

At present there is a gap in the literature around the admission criteria in aged care facilities for residents who are subject to homelessness [27]. This is partly due to the very few residential care options available for this cohort of residents [18]. In a series of scoping reviews investigating the needs for housing and potential solutions for older homeless people, Canham and Humphries [18, 55] discussed the Wicking project (Melbourne, Australia) [42, 56] as one of the few international programs providing intensive support and specialised case management in a similar facility to the one described in the present study. This gap in the literature highlights a more serious issue in the healthcare system where high care and palliative care are sporadically provided for people who are subject to homelessness [10, 21]. At present there is call for action in the literature about the need for the provision of specialised end of life care for homeless people [22, 57–63]. End of life care for homeless people frequently takes place in hostels, where staff are not trained to adequately assist residents [64] or in less frequent cases, in support homes [65].

### Perception of the impact of living and working in a purpose-built care home

Finally, this study adds to the literature reporting significant improvements in mental health, quality of life outcomes, and reduced acute care utilisation from providing housing stability for older people subject to homelessness [5, 44, 66, 67], all of which are consistent with our findings from the quantitative evaluation of this care home [5, 30, 44, 66, 67]. The qualitative aspects of the evaluation reported in this paper found that residents’ views on their experiences from living in the care for six months were positive, together with the perception that their quality of life had improved, even if not always combined with an improvement in their physical health [30]. The newly dignified way of living that the residents had experienced since moving into the care home provided them with a stability that, in turn, had an impact on their perceived mental health (less stressed and with a new sense of purpose). Dignity of risk also might explain why allowing a resident to refuse treatment can be considered more dignified than insisting on safety and why being allowed to take risks, in a care home environment, can enhance residents’ wellbeing [68]. Håkanson et al. [65] discuss the concept of ‘re-dignifying’ the person through interaction with staff that in the context of a care home for homeless residents, often becomes a family substitute as they are commonly estranged from their families [69].

The new sense of purpose that some of the residents reported since moving into the care home might be linked with living in a safe and supported environment where they were looked after medically and were assisted by nurses, care staff, spiritual care staff and volunteers that regularly interacted with them. This is also in line with the results of a ‘Housing First’ program in Canada [66] where improved housing stability among older homeless people resulted in improved mental health and quality of life outcomes.

### Limitations

This study has a number of limitations. The evaluation was conducted at one care home, located in Sydney, Australia. While the residents and staff were from a range of cultural and social backgrounds, the results may not be generalisable to the general population of older people subject to homelessness. While there are different factors that contribute to homelessness in older age [70], due to the study scope and the small sample size, it was not possible to do an in-depth exploration of these differences, and how these may have impacted on resident experiences living within the home. For example, future work could look at potential differences in experiences between older people who are homeless for the first time in older age, those who have experienced long-term homelessness throughout their life, those living at-risk of homelessness, and the particular experiences and needs of older women subject to homelessness.

### Conclusions and implications for practice

This study provides novel qualitative insights into how the lives of older people subject to homelessness with high care needs are affected by living in a specifically designed care home, and on some of the challenges faced by staff in working within and managing the home. Residents reported generally positive experiences from living within the care home, despite reporting instances of conflict, mostly with the behaviour of other residents. A central finding was that of the complex interplay between resident dependency and behaviours, referral pathways and stakeholder engagement, government funding models and requirements, staff training and wellbeing, and the need to meet operational viability. A key challenge for staff, especially those who had previous health and aged care experience, was the need to reconcile delivery of a trauma informed model of care that seeks to support collaboration, choice and empowerment, with their previous experience working within more traditional settings, highlighting that a significant gap in the healthcare system remains when it comes to the provision of high care for people subject to homelessness [71]. The development of similar services in the future should consider ongoing specialised training in supporting older people subject to homelessness. Based on the outcomes from this study, this training should have a specific focus on implementing a trauma-informed approach to aged care (to support identified histories e.g. addiction, family break up, traumatic childhoods), supporting mental health, and providing strategies to manage behavioural challenges that might be presented by residents. Future research could also explore specialised staff training through the inclusion of recovery support into the care model, where residents could also be supported through actively processing some of their past trauma.

Study outcomes have also highlighted the challenges around balancing the financial and social/organisational aspects of the admission criteria, which contributed to frustrations when stakeholder expectations were at variance to the care home staff. To overcome these challenges, there is a need for clearer communication with key stakeholders (e.g., hospitals and other homelessness services) around admission criteria, thereby facilitating the flow of more appropriate referrals, which in turn would help support occupancy and the operational and financial requirements of the home.

Finally, given that specialised services to support older people subject to homelessness with high-care needs are still in their infancy, it is suggested that future services should also consider adopting formal service evaluations, allowing the opportunity for continued knowledge sharing.

## Data Availability

The data that support the findings of this study are available from the corresponding author upon reasonable request and subject to ethical approvals.
